# The Role of AIRE Deficiency in Infertility and Its Potential Pathogenesis

**DOI:** 10.3389/fimmu.2021.641164

**Published:** 2021-02-19

**Authors:** Xueyang Zou, Yi Zhang, Xiaoya Wang, Rongchao Zhang, Wei Yang

**Affiliations:** Department of Immunology, College of Basic Medical Sciences, Jilin University, Changchun, China

**Keywords:** AIRE, infertility, ovary, uterine, testis, apoptosis in spermatogenesis, autoantibodies

## Abstract

The increasing number of patients with infertility is recognized as an emerging problem worldwide. However, little is known about the cause of infertility. At present, it is believed that infertility may be related to genetic or abnormal immune responses. It has long been indicated that autoimmune regulator (AIRE), a transcription factor, participates in immune tolerance by regulating the expression of thousands of promiscuous tissue-specific antigens in medullary thymic epithelial cells (mTECs), which play a pivotal role in preventing autoimmune diseases. AIRE is also expressed in germ cell progenitors. Importantly, the deletion of AIRE leads to severe oophoritis and age-dependent depletion of follicular reserves and causes altered embryonic development in female mice. AIRE-deficient male mice exhibit altered apoptosis during spermatogenesis and have a significantly decreased breeding capacity. These reports suggest that AIRE deficiency may be responsible for infertility. The causes may be related to the production of autoantibodies against sperm, poor development of germ cells, and abnormal ovarian function, which eventually lead to infertility. Here, we focus on the potential associations of AIRE deficiency with infertility as well as the possible pathogenesis, providing insight into the significance of AIRE in the development of infertility.

## Introduction

AIRE is a 545-amino-acid glycoprotein that was originally reported in 1997 (1) and has been mapped to regions of human chromosome 21q22.3 (1) and mouse chromosome 10 (2, 3). Since then, research on the immunologic function of AIRE has demonstrated its multiple functions. AIRE acts as a transcription factor and is predominantly expressed in mTECs (4), where it induces the ectopic expression of a variety of tissue-specific antigens (TSAs) (5) and hence is thought to contribute to the negative selection of self-reactive thymocytes, thereby maintaining central immune tolerance. In addition, AIRE protein has also been detected in multiple peripheral tissues and cell types, such as dendritic cells (DCs) (6). Peripheral AIRE-expressing DCs participate in the establishment of peripheral tolerance through clearance of autoreactive CD4^+^ T cells or inhibition of Th1, Th17, Th22, and TFH differentiation (7). In these respects, AIRE has emerged as one of the most critical actors in the establishment of self-immunological tolerance, which, as a result, prevents autoimmune disease.

Functional AIRE gene mutation causes autoimmune polyendocrinopathy-candidiasis-ectodermal dystrophy (APECED), the main clinical manifestation of which is severe multiple organ-specific autoimmune disorders in humans (8). Additionally, emerging evidence has demonstrated that AIRE deficiency is also related to infertility, one of the comorbidities associated with APECED. Infertility is regarded as a fairly common problem around the globe. After a period of 1 year of unprotected intercourse, ~15% of couples fail to conceive. The prevalence of infertility has increased noticeably in recent years because of delayed childbearing. Multiple factors are involved in the development of infertility. Female factors contribute to more than half of diagnosed cases of infertility, and the potential causes correlate very well with a variety of autoimmune disorders targeting the ovary directly or secondarily following autoimmune disease (9–12). Most female APECED patients with infertility could be explained by ovarian failure. In other cases, infertility is attributed to male factors. Testicular atrophy, hypogonadotrophy, azoospermia anti-sperm antibodies, and Leydig cell antibodies were significantly associated with infertility in male patients (13). Importantly, most of the available data suggesting a role for AIRE in infertility was deduced from mouse models lacking AIRE. An increasing number of infertility studies have highlighted the effect of AIRE deficiency, which has received relatively little attention in the public arena. In the near future, AIRE may become a focal point in the field of infertility. Thus, in this review, we specifically focus on the relationship between AIRE deficiency and female and male infertility in mice. In addition, we discuss the potential mechanism underlying the pathological link between AIRE loss and infertility.

## AIRE Deficiency is Responsible for Infertility

The important role of AIRE in central and peripheral tissues has received enormous attention over multiple decades. More recently, accumulating evidence has indicated that AIRE is also expressed in the ovary (14) and testis (15) except the immune system. Many studies have reported that AIRE deficiency may result in infertility in females or males, suggesting that the loss of AIRE is one of the most determinant factors of infertility.

### Female Infertility

In 1999, AIRE was first detected within the ovary in C57BL/6 mice by Northern blot and RT-PCR (14). Then, one set of pregnancy model studies demonstrated high levels of AIRE expression in the decidual basilis and mesometrial lymphoid aggregate of pregnancy (MLAp) in the uterus at gestational day (GD) 10.5 (16). Later, experimental data from Soumya et al. showed that AIRE expression is markedly upregulated at embryo implantation day 5 and is most prominent within the primary decidual zone (PDZ) of the uterus (17). This points to its possible function in the development of embryo implantation with the subsequent pregnancy success rate. This mechanism is also favored by the observation of significantly impaired embryo implantation as well as reduced fertility in AIRE-deficient female mice reported by Warren (18). In these senses, the loss of AIRE in mice may be responsible for female infertility. First, AIRE deficiency resulted in a decreased rate of pregnancy. At 6 weeks of age, although female mice either with or without AIRE deficiency have overtly normal ovaries and display essentially normal copulatory behavior, only half of AIRE-deficient females become pregnant after cohabitation with wild-type (WT) males (19), suggesting that the fertility of AIRE-deficient mice is compromised from an early reproductive age. Further decline in frequency of fertile females is observed with advancing age. Among the other half of AIRE-deficient mice that failed to deliver litters, 83% exhibited severely disrupted estrous cyclicity. All control females delivered litters, and none of them displayed impaired cyclicity (19). In addition, compared with WT females, AIRE-deficient mice show significantly decreased potential to deliver a second litter (20). Among all AIRE-deficient females, 80% failed to deliver pups (20). Finally, another line of evidence indicating a direct role of AIRE in fertility is that deletion of the AIRE gene results in a dramatic impairment of fetal development. When AIRE-deficient pregnant mice and WT controls were sacrificed on the 13th day of pregnancy, the majority of control females had normal fetuses in the horn, while all AIRE-deficient females experienced fetal loss (17). Overall, studies using these mouse models lacking AIRE point to a critical role played by AIRE in maintaining normal reproductive function, and female mice with deletion of AIRE suffer from high rates of infertility.

### Male Infertility

In the initial characterization of AIRE in the testis, AIRE mRNA was detectable, but protein could not be identified by Western blotting (15). It was not clear whether AIRE mRNA can effectively be translated into protein in the testis or in which cell type it is expressed. A subsequent study from Claudia et al. suggested that the use of polyclonal Abs may be one of the reasons for the discrepancies between mRNA and protein results. By using a monoclonal Ab, they found that testicular AIRE expression was specifically limited to early spermatocytes and spermatogonia and was not present in spermatids or Sertoli cells (21). Nonetheless, studies on the testis expression of AIRE vary considerably. The results of a recent finding supported the opposite fact that the AIRE protein is abundantly expressed in mouse testicular lysates at all evaluated stages of germ cell development, including in meiotic germ cells, primary spermatocytes, post meiotic cells, secondary spermatocytes and spermatids (22). Interestingly, a research group observed a gradual decreasing trend in AIRE expression in the testis with sexual maturity. RT-PCR analysis showed that AIRE mRNA is expressed at the highest level in the testes of male mice at the age of 1 week, which is markedly higher than the level at the age of 4 weeks. The levels of AIRE expression in the testes of 2-week-old and 3-week-old mice showed a 4- or 3-fold increase compared with that in 4-week-old testes (22). Notably, female mice failed to produce any litters after intercrossing with Aire-deficient males, indicating fertility defects. A recent study carried out on AIRE-deficient male models revealed that the fertilization success rate was markedly downregulated compared with that of WT controls (23), suggesting the decreased quality and oocyte fertilization capacity of sperm from AIRE-deficient mice, which further negatively impacts fertility in these mice.

## The Effect of AIRE Deficiency in Infertility

Since the depletion of AIRE is responsible for infertility, a large number of studies have attempted to quantify the effects of AIRE deficiency on infertility. The pathology of infertility in AIRE-deficient mice displays sex differences. The observation of impaired embryonic development together with the presence of follicular depletion and T cell inflammatory infiltrates in Aire-deficient female mice has been well-reported. Male mice lacking AIRE displayed reduced scheduled apoptosis in spermatogenesis. These results provide a potential explanation for the observed variations in female and male fertility.

### The Effect of AIRE Deficiency in Female Infertility

#### Reduced Embryonic Development

A recent experimental study favored delayed embryonic development as the major reason for infertility in females lacking AIRE. AIRE-deficient female mice and WT controls, 6 weeks old, were mated with fertile males, and heterozygous embryos were quantified by counting the numbers in the uteri of the females on GD 3.5. Although no apparent distinction in the numbers of embryos was seen between strains, the developmental stages of the embryos showed a significant difference. The presence of embryos at the blastocyst stage was observed in all WT dams, with the percentage of embryos reaching the blastocyst stage being 57–100%, whereas in four KO dams, blastocysts were found in only two of the dams, and within these, only 12.5 and 62.5% of embryos had grown to the blastocyst stage. In total, 80% of embryos from WT mice had grown to the blastocyst stage, while only 20% of embryos from AIRE-deficient mice reached this stage, a marked decrease (19). These results might reflect a direct role of AIRE in early embryogenesis. Interestingly, no difference was observed in the implantation rates of WT and AIRE-deficient dams at GD5.5, suggesting developmental delay in embryos rather than arrest. Consistent with this, GD3.5 embryos from AIRE-deficient dams cultured *in vitro* did not show extensive outgrowth of trophoblasts compared with the control (19). The slowed growth may have resulted in reduced developmental competence. Overall, these data indicated the pronounced role of AIRE depletion in reduced embryonic development, which could cause low fertility.

#### Ovarian Follicular Depletion

The ovaries are filled with follicles, which play an important role in oocyte maturation. Susmita et al. found the depletion of ovarian follicular reserves in female mice lacking AIRE, which displayed a failure to mate (20). Histological section analysis was used to quantify the follicular reserve in virgin AIRE-deficient female mice and BALB/c controls at 1, 4, 8, 12, 16, and 20 weeks of age. The loss of ovarian follicles was seen in approximately 30% of AIRE-deficient females at 8 weeks of age. Of the control females, none of the BALB/c mice exhibited follicular depletion. Later, a higher percentage of AIRE-deficient mice displayed follicular depletion with advancing age, ranging from 50 to 60% at the age of 12–20 weeks. However, of previously bred AIRE-deficient females at the same age, ~80% displayed complete ovarian follicular reserve loss (20). Apparently, pregnancy itself may further predispose these mice to ovarian follicular depletion. Moreover, as previously described by Susmita (20), 10 of 12 AIRE-deficient females failed to deliver either a first or second litter. The other two mice that could deliver a second litter also showed signs of depletion of follicular reserves despite possessing histologically normal ovaries, suggesting that AIRE deletion probably affects follicles at multiple stages. Furthermore, in transplantation experiments, ovaries transplanted from WT animals into AIRE-deficient females exhibited rapid depletion of ovarian follicles in recipients whose ovaries were similarly empty of oocytes (20), suggesting that follicular loss depended on factors extrinsic to the ovary. Taken together, these data strongly suggest that infertility in female mice lacking AIRE is correlated, at least in part, with progressive ovarian follicular depletion.

#### Infiltration of Inflammatory T Cell in Ovaries

Inflammation is considered a major causal factor related to reproductive dysfunction, including the most common causes of female infertility, such as pelvic inflammatory disease, obesity, polycystic ovary syndrome, endometriosis, and recurrent pregnancy loss (24). It is likely that a defect in AIRE negatively regulates fertility in female mice by mediating the inflammatory process, a notion supported by the observation of T cell inflammatory infiltrates in or around the ovaries of female mice lacking AIRE. Immunohistochemistry analysis of ovarian tissues revealed significantly increased expression of CD3, a marker of T lymphocytes, in AIRE-deficient mice compared with control mice. At 4 weeks of age, 57% of AIRE-deficient female mice had CD3^+^ T cell infiltration. The percentage of CD3-positive ovarian cells increased with advancing age in AIRE-deficient females and reached 96% by 20 weeks of age. It seems likely that these females would display follicular depletion later. Consistently, these T cells in the ovary resident to surround follicles, infiltrate the corpora lutea, or organize into aggregates. Only 3% of WT mice displayed detectable CD3^+^ T cells (20). Furthermore, Th1 cells appear to contribute to infertility in AIRE-deficient females by mediating ovarian infiltrates. Women with premature ovarian failure also display similar immune infiltrates (25). In summary, the depletion of AIRE might cause T cell inflammatory infiltration, which is a causative factor of infertility.

### The Effect of AIRE Deficiency in Male Infertility

In the last few years, several research groups have postulated that exquisitely coordinated interactions between Sertoli cells and germ cells are necessary for normal mature spermatogenesis. The ratio of germ cells in different developmental stages to Sertoli cells is believed to play an important role, as the apoptotic wave spares Sertoli cells. This process would further lead to permanent impairment of spermatogenesis, which would increase the prevalence of male infertility (26). Nevertheless, one recent report is not consistent with these results. Schaller and colleagues (21) propose that scheduled apoptosis of spermatogenesis is one of the critical events for genomic health. A decrease in scheduled apoptotic waves of germ cells has been demonstrated in AIRE-deficient male mice at 3 weeks of age. TUNEL assays showed that 1.6% of cells in WT mice were apoptotic, while AIRE-deficient mice displayed a 25% reduction compared with the control. The most affected apoptotic cells in AIRE-deficient males are spermatocyte II cells, which exhibit only half of the apoptosis observed in WT mice. Interestingly, sporadic apoptosis in adult mice at the age of 3 months was upregulated in AIRE-deficient mice, most likely due to mutated AIRE resulting in proliferation and further differentiation of spermatocytes, thereby leading to more cell death. This line of evidence implies an association between scheduled and sporadic apoptotic processes and suggests that scheduled apoptosis provides a counterselection mechanism that keeps the germline stable. On the other hand, another plausible explanation is that male lacking AIRE with high incidence (71%) of autoimmune prostatitis could be responsible for infertility (27). Motrich et al. (28) reported that male with prostatitis have upregulated level of TNF-α, IFN-γ and IL-1 in seminal plasma, these inflammatory cytokines have detrimental effect on sperm motion and viability. It is thus possible that the deletion of AIRE in male could also negatively affect the semen and sperm quality. Collectively, these findings indicate that the definite consequence of AIRE deficiency in the testes is a reduction in germ cell apoptosis as well as semen and sperm quality that results in reduced fertility.

## Molecular Mechanisms Underlying AIRE Deficiency in Infertility

Several studies in mice have been presented to explain the molecular mechanism by which AIRE deficiency results in defects in fertility. These downstream molecules were supported by several lines of evidence, and a major portion of them include decidual markers and autoantibodies against ovaries and testes.

### The Molecular Mechanisms of AIRE Deficiency in Female Infertility

#### Bmp2, Bmp4, Igfbp1, and Hoxa10

As mentioned above, failure of embryo implantation in AIRE-deficient female mice is one of the major reasons for infertility. Females with implantation failure have shown impaired decidualization, a vital process in the early pregnancy period, that further negatively regulates embryonic survival (29). One morphometric analysis identified smaller implantation sites, compromised primary decidual zones, and reduced embryo sizes in AIRE-deficient female mice compared to WT control mice. Decidual markers; bone morphogenetic protein-2,4 (Bmp2, Bmp4) (30), which are focal to decidual reprogramming during early pregnancy; insulin-like growth factor-binding protein 1 (Igfbp1); and homeobox A10 (Hoxa10) (31) are conserved genes that regulate progesterone-induced regional decidualization in implantation and play key roles in the uterus and during pregnancy. AIRE is well-known to influence the transcription of these genes in the endometrial stromal cells by binding to their promoters. The mRNA expression of Bmp2 and Bmp4 in AIRE-deficient uteri was reported to be significantly abolished. Western blotting analysis suggested a significant downregulation of these factors in AIRE-silenced uteri, and immunohistochemistry results confirmed this effect *in vivo*. Igfbp1 and Hoxa 10 are reduced in AIRE-deficient uteri at both the mRNA and protein levels (17). Overall, these results support the idea that the key molecules downregulated by AIRE defects are involved in decidualization-mediated loss in the decidual reaction, which is responsible for the smaller implantation sites and decreased embryonic growth.

#### Anti-ovarian Antibodies

It has been well-established that ovarian autoimmunity is one of the causes of female infertility. Females lacking AIRE develop autoimmune oophoritis, whose manifestations include histologic disease and frequent oligoclonal autoantibodies to the ovary (32). In addition, the presence of anti-ovarian antibodies (AOAs) has been shown to influence the development of eggs and embryos as well as contribute to implantation failures (33). All these findings point to the possible effect of AIRE defects on the production of anti-ovarian antibodies. Further support has come from the observations of autoantibodies in female mice lacking AIRE, as assessed by Cheng (25). AIRE-deficient mice ovulated degenerated oocytes somewhat frequently (31%), whereas none of the control mice did. Sixty percent of AIRE-deficient female mice at 6–8 weeks of age produced serum autoantibodies against the ovary and oocytes (20). Moreover, autoantibody immunoreactivity suggests a wide array of autoreactive targets, including stromal cells, luteal cells, and oocytes (18). Interestingly, these targets may vary according to whether the female has pregnancy signs and symptoms. Western immunoblotting analysis showed that antigen molecules such as zona pelucida (ZP)2 and ZP3 are oocyte-specific and are detectable in non-pregnant AIRE-deficient female serum but not in pregnant AIRE-deficient or WT females (5, 34), while another molecule, betaine-homocysteine methyltransferase (BHMT), is only present in pregnant AIRE-KO mice. These factors are all potentially ovarian targets that can affect both antibody production and infertility (18). In females, hypogonadism relative to APECED is probably caused by autoantibodies against steroidogenic enzymes, in addition to targeting of ovary-specific antigens. These include Cytochrome P450 Family 11 Subfamily A Member 1 (CYP11A1) and Cytochrome P450 17alpha-hydroxylase/17,20-lyase (CYP17), which are also observed in the adrenal gland (35, 36). These studies confirm the roles of antiantibodies in the pathogenesis of infertility in AIRE-deficient females.

### The Molecular Mechanisms of AIRE Deficiency in Male Infertility

#### TGM4

Transglutaminase 4 (TGM4), a prostatic secretory enzyme, is identified as a male-specific autoantigen. Autoantibodies against TGM4 can be found in the majority of adult male patients with APECED but were not detected in young males, which suggests a potential effect of AIRE mutation in mediating the secretion of TGM4 autoantibodies in males. Consistent with these findings, in a mouse model, TGM4 autoantibodies were detectable in all AIRE-deficient males, whereas this kind of antibody was not detected in any of the AIRE-deficient female or WT mice (37). On the other hand, TGM4 most likely plays a key role in male reproductive physiology through various mechanisms. TGM4 is well-known to be a main regulator of semen viscosity and induces viscosity and coagulation of semen by cross-linking gel-forming proteins (38, 39). It can also regulate the modification of the sperm surface, mediating the capacitation of sperm; thereby, TGM4 further appears to be critical for sperm to acquire the features of a fully differentiated fertile cell (40). These important functions are severely impaired in TGM4 knockout mice, which display severely reduced male fertility. AIRE-deficient mice with TGM4 autoantibodies exhibited failure of TGM4 secretion and appear to show the same fertility defects as TGM4-knockout mice. Overall, male infertility could be the result of increased expression of TGM4 autoantibodies mediated directly or indirectly by AIRE defects.

#### SVS2

Seminal vesicle secretory protein 2 (SVS2), a major component of seminal vesicle secretions expressed in the prostate gland, is critical for successful internal fertilization through protection of the sperm membrane against a uterine immune attack (41). Male mice lacking SVS2 protein consistently display reduced fertility efficiency. Hou et al. reported AIRE is required for SVS2 expression in thymus, the deletion of AIRE significantly reduced the thymic expression of the *SVS2* gene (27), which will further impair the formation of copulatory plugs in natural mating may result in male infertility. The sperm ejaculated from male mice lacking SVS2 is considered to undergo ectopic activation in the uterine cavity, effectively resulting in failure to entry into the oviduct (42). On the other hand, the deletion of SVS2 in mice could disturb sperm plasma membrane, presumably leading to intrauterine sperm death (42). Therefore, SVS2 impairment is closely associated with infertility mediated by AIRE loss.

## Conclusion and Perspectives

**Figure 1:**
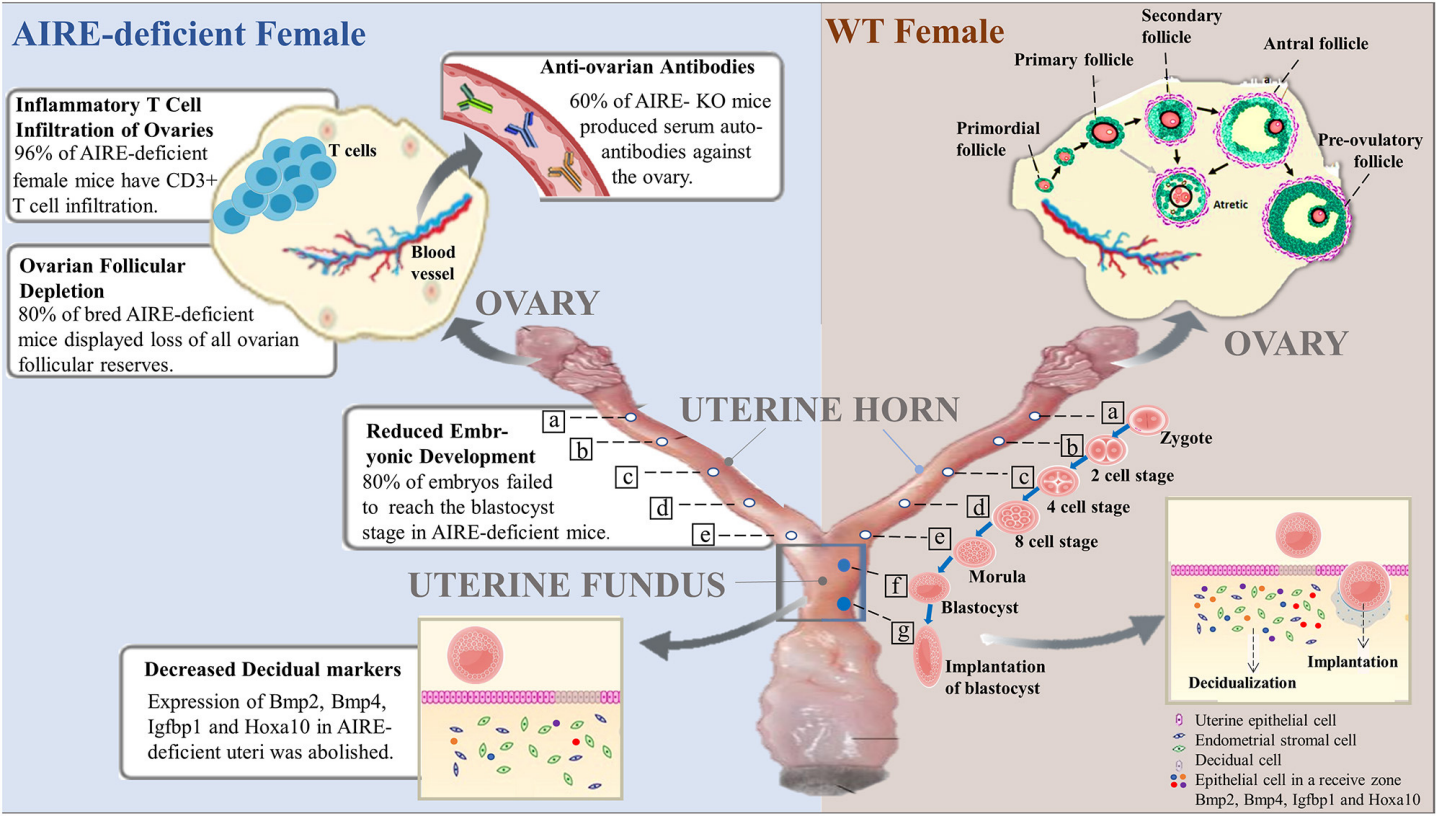
Roles of AIRE deficiency in the pathogenesis of female infertility. In wild-type female mice, follicles at the primordial stage with activation develop into preovulatory follicles and are finally released from the ovary to become fertilized. The fertilized egg (zygote) moves to the uterus to become a blastocyst that implants in the wall of the uterus. Notably, decidualization, the rapid proliferation of endometrial stromal cells into decidual cells, is required for implantation. Successful implantation is considered to be essential for pregnancy. However, female mice lacking AIRE exhibit marked defects in ovarian follicles, blastocysts and decidualization. Additionally, AIRE deficiency may induce the production of autoantibodies against the ovary and infiltration of inflammatory T cells, resulting in pathological alterations and female infertility.

**Figure 2:**
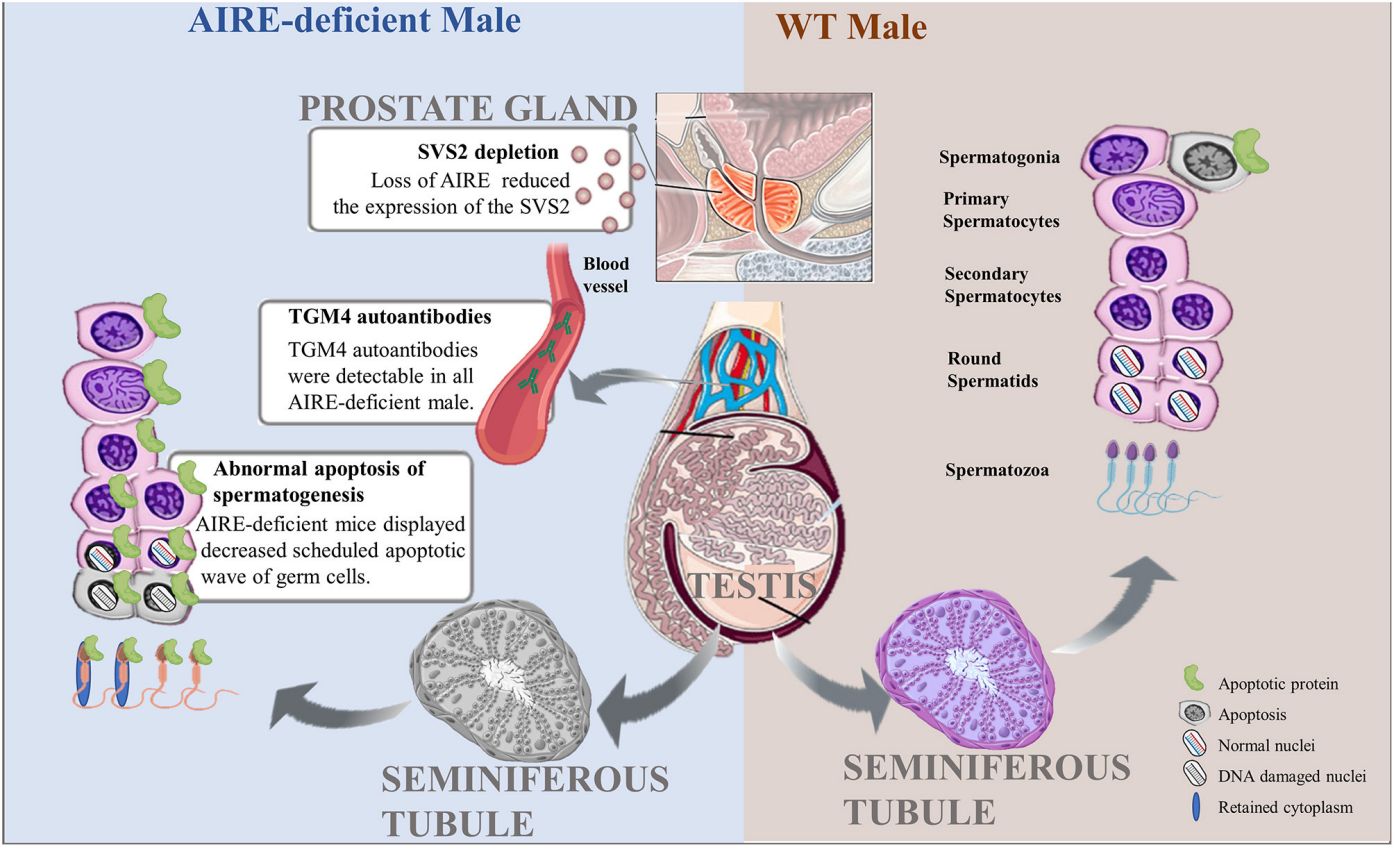
Pathological roles of AIRE deficiency in the pathogenesis of male infertility. Scheduled germ cell apoptosis is critical for spermatogenesis in male testes. AIRE-deficient males fail to display the normal scheduled apoptotic wave of germ cells, possibly leading to nuclear DNA damage or cytoplasmic abnormalities and further producing abnormal spermatozoa. In addition, AIRE defects increase the expression of TGM4 autoantibodies in serum and reduced the secretion of SVS2 from prostate gland, resulting in male infertility.

Researchers have improved their understanding of the association between AIRE deficiency and infertility. Loss of AIRE in females results in a reduced rate of pregnancy and impaired fetal development. These effects are most likely a result of decreased oocyte quality due to the presence of anti-ovarian autoantibodies, wholesale follicular depletion, a direct effect of AIRE on embryonic development, or the asynchrony between embryonic development and uterine implantation, further leading to embryonic demise shortly after implantation. These are all highly suggestive of the effects of the loss of AIRE on oocyte and embryonic health (Figure 1). Moreover, AIRE is detectable in the testis, and in male mice lacking AIRE expression, the scheduled apoptotic wave of germ cells, which is necessary for normal spermatogenesis and maturation, is decreased. Aire-deficient mice with TGM4 autoantibodies lacked TGM4 production, displaying severely decreased male fertility (Figure 2). Taken together, these studies have firmly established the importance of AIRE in healthy pregnancy. AIRE deficiency negatively impacts fertility in mice.

However, there are still some big challenges ahead of us. Studies from Jasti et al. identified placental syncytiotrophoblasts as autoimmune targets in the presence of autoantibodies, suggesting that immune-mediated pregnancy loss may be a result of AIRE deficiency. However, future research should address the possible mechanism underlying the role of placental syncytiotrophoblasts in infertility. Furthermore, in addition to autoantibody induction by AIRE deficiency causing spermatogonial apoptosis, the existence of mechanisms distinct from AIRE still needs to be explored. A limitation of the current literature is that the identity of the antigenic targets in the ovary and other tissues of the reproductive tract have not been revealed; more research is needed on this topic. Future studies will also reveal cell types in addition to T cells that regulate autoimmune damage in the ovary. In addition, a variety of reproductive antigens are detectable in mTECs, including limited expression in organs of the female and male reproductive tracts. It is not clear whether autoreactive T cells escape negative selection in the thymus due to the abnormal expression of autoantigens in reproductive organs with AIRE deficiency. This mediates the autoimmune response to the ovary or testis, subsequently resulting in the development of infertility. Moreover, AIRE-deficient mice often suffer from autoimmune disease such as lupus (43), rheumatoid arthritis (44) or type I diabetes (45) but may develop inflammation in prostate that commonly induce the risk for infertility. Additional research is required to strengthen the potential suggestions that AIRE deficiency may indirectly impair fertility by mediating those disease. Therefore, we need to further study the mechanisms involved in female and male infertility associated with AIRE deficiency.

## Author Contributions

WY conceptualized the study. XZ drafted the manuscript. YZ, XW, and RZ conducted the literature review. All authors contributed to the article and approved the submitted version.

## Conflict of Interest

The authors declare that the research was conducted in the absence of any commercial or financial relationships that could be construed as a potential conflict of interest.
